# Association Mapping for Yield Attributing Traits and Yellow Mosaic Disease Resistance in Mung Bean [*Vigna radiata* (L.) Wilczek]

**DOI:** 10.3389/fpls.2021.749439

**Published:** 2022-01-17

**Authors:** Versha Rohilla, Rajesh Kumar Yadav, Atman Poonia, Ravika Sheoran, Gita Kumari, P. S. Shanmugavadivel, Aditya Pratap

**Affiliations:** ^1^Department of Genetics and Plant Breeding, Chaudhary Charan Singh Haryana Agricultural University, Hisar, India; ^2^ICAR-Indian Institute of Pulses Research, Kanpur, India

**Keywords:** association mapping, yield attributing traits, *Vigna radiata*, SSR, linkage disequilibrium, MLM

## Abstract

Mung bean [*Vigna radiata* (L.) Wilczek] is an important short-duration grain legume widely known for its nutritional, soil ameliorative, and cropping system intensification properties. This study aims at evaluating genetic diversity among mung bean genotypes and detecting genomic regions associated with various yield attributing traits and yellow mosaic disease (YMD) resistance by association mapping. A panel of 80 cultivars and advanced breeding lines was evaluated for 10 yield-related and YMD resistance traits during *kharif* (monsoon) and summer seasons of 2018–2019 and 2019–2020. A total of 164 genome-wide simple sequence repeat (SSR) markers were initially screened, out of which 89 were found polymorphic which generated 317 polymorphic alleles with an average of 3.56 alleles per SSR locus. The number of alleles at each locus varied from 2 to 7. The population genetic structure analysis grouped different genotypes in three major clusters and three genetically distinct subpopulations (SPs) (i.e., SP-1, SP-2, and SP-3) with one admixture subpopulation (SP-4). Both cluster and population genetic structure analysis categorized the advanced mung bean genotypes in a single group/SP and the released varieties in other groups/SPs, suggesting that the studied genotypes may have common ancestral history at some level. The population genetic structure was also in agreement with the genetic diversity analysis. The estimate of the average degree of linkage disequilibrium (LD) present at the genome level in 80 mung bean genotypes unveiled significant LD blocks. Over the four seasons, 10 marker-trait associations were observed significant for YMD and four seed yield (SY)-related traits *viz*., days to flowering, days to maturity, plant height, and number of pods per plant using the mixed linear model (MLM) method. These associations may be useful for marker-assisted mung bean yield improvement programs and YMD resistance.

## Introduction

Mung bean [*Vigna radiata* (L.) Wilczek], also known as green gram, is an annual herbaceous self-pollinated pulse crop having diploid chromosome number 2*n* = 2*x* = 22 (Karpechenko, 1925). It has a small genome size of 543 Mbs (Kang et al., 2014), which makes it a valuable model for advancing the understanding of genetic diversity and genome evolution. It is an important food legume in Asia and parts of Africa and America (Pratap et al., 2020). As a short-duration crop (55–70 days from sowing to maturity), it can be grown across seasons in varying cropping systems and crop rotations (Malik, 1994). It plays an important role in sustaining soil fertility by improving the physical and biological properties of the soil. In association with *Bradyrhizobium* bacteria, it fixes atmospheric nitrogen in the soil (Joshi et al., 2003). Mung bean is highly nutritious and an inexpensive source of easily digestible high-quality protein, amino acids, lipids, fat, fiber, ash, and carbohydrates and provides 334–344 kcal energy (Srivastava and Ali, 2004; Day, 2013; Choudhary and Suri, 2014; Singh et al., 2018). Besides, mung bean seeds have no anti-nutritional factors such as trypsin inhibitors, phytohemagglutinin, and tannin (Chen et al., 2003). Currently, the realized average productivity of mung bean is well below the economic level. The major reasons for stagnancy in its productivity are limited genetic variability, negative impact of high genotype × environment interaction (GEI), and susceptibility of the existing cultivars to various biotic and abiotic stresses, ultimately leading to yield instability (Chauhan et al., 2010; Pratap et al., 2019a). Modern crop breeding has further resulted in an increase in the genetic uniformity among the mung bean cultivars leading to further erosion of the genetic diversity.

Assessment of genetic diversity is a step of paramount importance and is a prerequisite for improvement in any crop. The estimation of genetic diversity is valuable in the selection of diverse and compatible parental genotypes. This helps to generate segregating progenies with maximum genetic variability and also in the introgression of desirable traits from diverse or wild germplasm into the commercial cultivars to broaden their genetic base (Barrett and Kidwell, 1998; Saravanakumar et al., 2004; Sangiri et al., 2007). The most important agronomic and economic trait in crop plants is yield, which is a function of multiple interacting component traits, controlled by multiple loci with a largely ambiguous genetic basis. To launch a breeding program for the improvement of plant genotype with a desirable combination of traits, complete information regarding the association of these traits with yield as well as detailed information on the genetic mechanism controlling various traits is important.

Molecular studies provide more reliable data than morphological and physiological data (Rahman et al., 2011) owing to the lack of environmental influence. DNA markers such as restriction fragment length polymorphism (RFLP), amplified fragment length polymorphism (AFLP), inter simple sequence repeat (ISSR) and single nucleotide polymorphisms (SNPs) have commonly been used for genetic diversity studies in plants. Among these, SSR markers are reported to be highly reliable due to their high degree of polymorphism, multi-allelic nature, reproducibility, codominance, locus specificity, abundance, and capacity of wide genome coverage (Powell et al., 1996) when compared with other DNA markers. These have been widely used in various crop species as potent tools for evaluation of genetic diversity (Somta et al., 2008), quantitative trait locus (QTL) mapping, genome-wide association study (GWAS) (Bohra et al., 2014), and marker-assisted selection (MAS) (Kumar et al., 2011; Pratap et al., 2017). Association analysis is a high-resolution method for genetic mapping using existing germplasm and their phenotypic information for the trait concerned (Flint-Garcia et al., 2003) and helps to understand the genetic basis of a complex trait like yield. It permits a survey of a wide range of alleles at each locus, detection of marker-trait associations at the whole genome level, and identification of elite alleles for significantly associated loci. Marker-trait association study has the advantage over conventional QTL mapping (Atwell et al., 2010) since it considers natural populations with more recombination events and mutations which might have occurred over multiple generations. On contrary, QTL mapping uses constructed biparental mapping population with limited recombination allowing detection of QTL in limited resolution. This creates a hindrance in the implementation of MAS in breeding programs, especially where linkage drag is a problem. Therefore, association study offers a higher mapping resolution of traits (Addington et al., 2011) and can overcome hindrance in the adoption of MAS in breeding programs (Mackay and Powell, 2007). This study aims to evaluate the genetic diversity and marker-trait associations in a panel of commercial mung bean cultivars and advanced breeding lines using SSR markers for genetic dissection of important SY-related traits along with yellow mosaic disease (YMD) resistance in order to expedite genetic improvement.

## Materials and Methods

### Plant Materials

The plant materials for this study comprised 80 diverse mung bean genotypes including 46 released cultivars recommended for cultivation in different agro-climatic zones in India and 34 advanced breeding lines developed at Chaudhary Charan Singh Haryana Agricultural University (CCS HAU), Hisar, India. The salient features and pictorial representations of the released cultivars are available elsewhere (Pratap et al., 2019b; Project Coordinator’s Report, 2020), whereas the advanced breeding lines are currently at different stages of multilocation evaluation for the possible release of the best ones as commercial cultivars.

### Phenotypic Evaluation

The genotypes were evaluated for yield traits and reaction to YMD caused by *Mung bean yellow mosaic India virus* (MYMIV, identity of causal virus established in other studies) in four seasons under field conditions during *Kharif* (Monsoon) and summer seasons of 2018–2019 and 2019–2020 at the Pulses Research Area of the Department of Genetics and Plant Breeding, CCS Haryana Agricultural University, Hisar, which is situated at a latitude of 29°10′N, 75°44′E, 215.2 m above msl. Each genotype was sown in a plot of three rows of 4 m length in two replications following a randomized complete block design. All the recommended agronomic practices for the experimental location were adopted to raise a robust crop. The genotypes were observed for yield-related traits *viz*., days to flowering (DF), days to maturity (DM), plant height (PH) in cm, pod length (PL) in cm, 100-seed weight (SW) in g, reaction to YMD, number of branches (NB) per plant, number of pods (NP) per plant, number of seeds (NS) per pod, and SY per plant in g. All these quantitative traits were measured in each plot on five randomly selected plants. Disease scoring for the YMD was performed 45 days after sowing (DAS) following Ahmed (1985) on a 0–9 scale (Supplementary Table 1). The correlation, mean values, SE, SD, and range were estimated for all the quantitative characters using IBM SPSS version 26.1 software.

### Genotyping the Mapping Panel

Young leaves were collected from all mung bean genotypes at the two-leaf stage for total genomic DNA extraction using the cetyltrimethylammonium bromide (CTAB) method as suggested by Saghai-Maroof et al. (1984) with minor adjustments. Extracted DNA quality was estimated by agarose gel electrophoresis (0.8%), and the quantity of DNA was determined using a NanoDrop spectrophotometer. Each DNA sample was normalized to a concentration of 50 ng/μl for use in PCR. PCR amplifications were carried out with 15 μl reaction mixture including 10× *Taq* buffer with 15 mM of MgCl_2_, 2.5 mM of dNTPs, 1 U of *Taq* DNA polymerase (GeNei Bangalore), 50 ng of template DNA, and 10 μmol of forward and reverse primers [Integrated DNA Technologies (IDT), Inc., United States] in an Applied Biosystem Thermocycler. The amplification conditions were programmed as initial denaturation at 94°C for 4 min followed by 35 cycles of denaturation at 94°C for 1 min, primer specific annealing at 45–55°C for 1 min, primer extension at 72°C for 1 min, and final extension at 72°C for 7 min. PCR products were resolved by using 3% agarose gel electrophoresis in 1× TBE buffer. Fragments were visualized under UV trans-illuminator and documented using BIO-RAD Gel Doc™ XR, United States, and alleles from each genotype were scored manually. A total of 164 SSRs from different *Vigna* species, namely, adzuki bean (Wang et al., 2004), common bean (Blair et al., 2013), cowpea (Li et al., 2001), and mung bean (Kumar et al., 2002; Somta et al., 2009; Pratap et al., 2016; Suman et al., 2019; Singh et al., 2020) used in this study are listed in Supplementary Table 2.

### Statistical Analysis

Statistical analysis such as mean, range, two-way ANOVA (Panse and Sukhatme, 1964), genotypic coefficient of variance (GCV), phenotypic coefficient of variance (PCV), broad-sense heritability, and genetic advance as percentage of mean was calculated for the 10 studied traits using INDOSTAT software^1^.

### Genetic Diversity Analysis

The allelic data of 89 polymorphic SSRs were scored in the form of base pairs (bp) and subjected to statistical analysis using GenAlEx version 6.51b2 to calculate the total number of alleles (Na), effective allele frequency (Ne), Shannon information index (*I*), observed heterozygosity (Ho), expected heterozygosity/genetic diversity (He), genetic differentiation indices, pair-wise population Nei genetic identity, and analysis of molecular variance (AMOVA) (Peakall and Smouse, 2006). The polymorphic information content (PIC) was calculated following Botstein et al. (1980) as PIC = 1 − Σ (*P*_ij_)^2^, where, *P*_ij_ denotes the frequency of *i*th allele of a *j*th locus summed across all alleles revealed by *j*th locus primer. Genotypic data of 89 polymorphic markers were used to generate distance-based weighted neighbor-joining (WNJ) dendrogram tree using DARwin 6^2^. The codominant allelic data were run at 30,000 bootstraps to draw the phylogenic tree and later, it was used as the robust signal for explaining the genetic diversity of released and advanced genotypes of mung bean.

### Population Structure Analysis

Population structure and the number of subpopulations (SPs) were determined using STRUCTURE software version 2.3.4 (Pritchard et al., 2000; Falush et al., 2007). The admixture model and correlated allele frequency model were selected to estimate the number of subgroups present in the association panel. Initially, 10 runs for the value of *K* ranging from 2 to 10 were conducted with a burn-in period of 100,000 followed by 200,000 Markov Chain Monte Carlo (MCMC) iterations. Then, the STRUCTURE HARVESTER web version 0.6.94 tool was used for obtaining the optimum *K* value determined by plotting the LnP (D) value against *K* (Earl and vonHoldt, 2012) which is based on the approach of Evanno et al. (2005).

### Association Analysis

Association analysis was conducted to reveal the marker-trait association using TASSEL software version 2.1 (Bradbury et al., 2007). General linear model (GLM) with Q matrix generated through STRUCTURE and mixed linear model (MLM) with kinship matrix (K) generated through TASSEL along with the Q matrix were used to extract information on the association of the markers with YMD and yield-related traits. The QQ plot was generated using R package (qqman).

## Results

### Genetic Variability and Correlation

ANOVA revealed highly significant mean squares for all the traits across four environments *viz*., *Kharif* (2018), *Kharif* (2019), Summer (2019), and Summer (2020) as well as in *Kharif* and Summer pooled over environments (Supplementary Table 3). Very less difference between PCV and GCV estimates was observed (Supplementary Tables 4a–c), and the GCV and PCV were categorized as low (<10%), moderate (10–20%), and high (>20%). Among the studied traits, YMD (41.03 and 43.39%) and the NB per plant (21.68 and 22.05%) had high GCV and PCV, respectively. High heritability (>60%) was recorded for all the traits with ranging from 97.69% in seed size to 65.95% for the NS per pod. The magnitude of genetic advance as percentage of mean was high (>20%) for YMD (79.89%), NP (43.91%), and SS (24.84%). Moderate genetic advance (10–20%) was observed for PH (18.65%), SY (17.60%), NP (16.01%), and PL (15%), whereas low genetic advance (<10%) was recorded for DM (7.56%), DF (6.13%), and NS (5.94%). High GCV, heritability, and genetic advance were observed for YMD and NP, while low GCV, high heritability, and low genetic advance were recorded for DF, NS, and DM.

Correlation of traits estimated using the pooled phenotypic data of all seasons revealed that the SY was positively associated with PL, NB, NP, and NS and negatively associated with YMD. DF showed a positive correlation with DM, PH, and YMD. DM showed a positive association only with DF. PH exhibited a significant positive association with DF, YMD, and NB. A positive correlation of PL was observed with SW, NS, and SY, whereas NB showed a significant positive association with PH, NP, and SY. SW was found positively correlated with PL and negatively correlated with PH and NB. YMD was observed to be positively associated with DF and PH, whereas negatively correlated with PL, NP, NS, and SY (Table 1).

**TABLE 1:** Correlation coefficients for various quantitative characters in mung bean.

	DF	DM	PH	PL	SW	YMD	NB	NP	NS	SY
DF	1									
DM	0.492**	1								
PH	0.353**	0.095	1							
PL	−0.230*	–0.048	–0.08	1						
SW	–0.174	–0.078	−0.332**	0.739**	1					
YMD	0.223*	0.030	0.254*	−0.385**	–0.194	1				
NB	0.121	–0.095	0.276*	−0.227*	−0.422**	–0.024	1			
NP	–0.182	–0.155	–0.183	0.100	–0.042	−0.689**	0.418**	1		
NS	–0.151	0.046	0.083	0.525**	0.166	−0.493**	0.108	0.342**	1	
SY	–0.152	–0.045	–0.098	0.305**	0.056	−0.737**	0.315**	0.708**	0.507**	1

*5% level of significance; **1% level of significance.

*DF, days to 50% flowering; DM, days to maturity; PH, plant height; PL, pod length; SW, 100-seed weight; YMD, yellow mosaic disease; NB, number of branches per plant; NP, number of pods per plant; NS, number of seeds per pod; SY, seed yield per plant.*

### Allelic Diversity

A total of 89 polymorphic SSRs were used to assess the genetic diversity among released cultivars and advanced breeding lines of mung bean. Most of the primer pairs amplified with varying allele sizes and ranged between 100 and 310 bp. All the polymorphic primer pairs generated 317 polymorphic alleles with an average of 3.56 alleles per SSR locus. The number of alleles at each locus (Na) varied from 2 (BMD-18, SSR-1AC127, SSR-1AC188, GMES1823, PVag003, PVag005, VR039, CP00361, CP5096, CEDG15, CEDG24, CEDG60, CEDG70, CEDG116, CEDG290, DQ9393, DQ469293, MBSSR008, PvM22, VM27, VR023, and VR032) to 7 (BM146, CEDG115, and GMES035). The number of effective alleles varied from 1.02 (CEDG290 and VR023) to 4.49 (BM146) with an average of 1.82. Shannon’s information index value varied from 0.07 to 1.63. The fixation index ranged from −0.93 to 1, and total 80 SSR loci showed the fixation index value 1. Heterozygosity was observed in nine SSR loci which ranged from 0.01 (CEDG41) to 0.97 (BMD-26) with an average of 0.05. The expected heterozygosity ranged from 0.02 (CEDG290 and VR023) to 0.78 (BM146) with an average of 0.38. The PIC value of SSRs varied from 0.02 (CEDG290 and VR023) to 0.96 (CEDG305) with an average of 0.43. The maximum PIC value was recorded for the marker CEDG305 (0.96) followed by DMBSSR080 (0.95), X62 (0.93), CEDG147 (0.93), DMSSR199 (0.91), and CP10667 (0.90) (Table 2).

**TABLE 2:** Details of polymorphic markers along with their allelic diversity and PIC.

Locus	Na	Ne	*I*	Ho	He	uHe	*F*	PIC
BM212	4.000	1.328	0.519	0.000	0.247	0.248	1.000	0.247
BM1	5.000	4.227	1.508	0.000	0.763	0.768	1.000	0.832
BM146	7.000	4.494	1.631	0.000	0.778	0.782	1.000	0.793
BMD-12	6.000	3.661	1.419	0.000	0.727	0.731	1.000	0.727
BMD-23	4.000	2.156	0.880	0.000	0.536	0.540	1.000	0.537
BMD-48	4.000	1.397	0.572	0.000	0.284	0.286	1.000	0.286
BMD-5	3.000	1.469	0.597	0.113	0.319	0.321	0.648	0.319
BMD-29	3.000	1.838	0.758	0.000	0.456	0.459	1.000	0.525
BMD-35	3.000	1.253	0.391	0.000	0.202	0.203	1.000	0.202
BMD-47	3.000	1.958	0.838	0.463	0.489	0.492	0.055	0.499
BMD-13	3.000	1.354	0.490	0.000	0.261	0.263	1.000	0.277
BMD-6	5.000	2.401	1.044	0.000	0.583	0.587	1.000	0.846
BMD-18	2.000	1.161	0.266	0.000	0.139	0.140	1.000	0.144
BMD-31	5.000	1.409	0.650	0.000	0.290	0.292	1.000	0.292
BMD-26	3.000	2.023	0.726	0.975	0.506	0.509	–0.928	0.506
CEDG115	7.000	3.313	1.387	0.000	0.698	0.703	1.000	0.879
CEDG147	5.000	2.658	1.180	0.000	0.624	0.628	1.000	0.913
CEDG220	6.000	1.407	0.656	0.000	0.289	0.291	1.000	0.297
CEDG244	6.000	1.527	0.749	0.000	0.345	0.347	1.000	0.345
CEDG254	5.000	3.302	1.339	0.000	0.697	0.702	1.000	0.720
CEDG256	3.000	2.696	1.036	0.825	0.629	0.633	–0.311	0.660
CEDG293	5.000	1.544	0.685	0.000	0.353	0.355	1.000	0.353
CEDG295	5.000	2.297	0.970	0.700	0.565	0.568	–0.240	0.572
CEDG296	6.000	1.665	0.860	0.000	0.399	0.402	1.000	0.415
CEDG305	5.000	2.143	1.067	0.000	0.533	0.537	1.000	0.956
CEDG048	3.000	1.701	0.720	0.000	0.412	0.415	1.000	0.412
CEDG053	3.000	1.414	0.509	0.000	0.293	0.295	1.000	0.293
CEDG071	4.000	2.546	1.067	0.000	0.607	0.611	1.000	0.705
CEDG073	3.000	1.594	0.628	0.000	0.373	0.375	1.000	0.373
CEDG088	6.000	2.775	1.220	0.000	0.640	0.644	1.000	0.890
CEDGAT009	5.000	3.604	1.362	0.000	0.723	0.727	1.000	0.773
CP1038	5.000	3.397	1.308	0.000	0.706	0.710	1.000	0.710
CP10667	4.000	2.982	1.227	0.000	0.665	0.669	1.000	0.902
DMSSR001	4.000	2.402	1.089	0.413	0.584	0.587	0.293	0.589
DQ345305	5.000	1.262	0.481	0.000	0.208	0.209	1.000	0.208
SSR-1AC127	2.000	1.190	0.297	0.000	0.160	0.161	1.000	0.167
SSR-1AC188	2.000	1.051	0.117	0.000	0.049	0.049	1.000	0.049
GMES162	3.000	1.569	0.635	0.000	0.363	0.365	1.000	0.398
GMES1823	2.000	1.311	0.400	0.000	0.237	0.239	1.000	0.237
GMES035	7.000	4.020	1.580	0.000	0.751	0.756	1.000	0.753
PVag003	2.000	1.161	0.266	0.000	0.139	0.140	1.000	0.144
PVag005	2.000	1.536	0.533	0.000	0.349	0.351	1.000	0.349
PVat001	3.000	1.569	0.635	0.000	0.363	0.365	1.000	0.398
PvM03	3.000	2.256	0.884	0.938	0.557	0.560	–0.684	0.561
PvM13b	5.000	1.972	0.936	0.000	0.493	0.496	1.000	0.538
SSR1AC-177	4.000	1.977	0.885	0.000	0.494	0.497	1.000	0.498
VR013	5.000	2.707	1.222	0.000	0.631	0.635	1.000	0.693
VR015	4.000	1.428	0.579	0.000	0.300	0.302	1.000	0.300
VR016	5.000	2.126	1.023	0.000	0.530	0.533	1.000	0.560
VR037	3.000	1.164	0.314	0.000	0.141	0.142	1.000	0.144
VR021	3.000	1.481	0.547	0.000	0.325	0.327	1.000	0.360
VR039	2.000	1.105	0.199	0.000	0.095	0.096	1.000	0.098
VrD1	3.000	2.506	0.994	0.000	0.601	0.605	1.000	0.864
X49	3.000	2.477	1.000	0.000	0.596	0.600	1.000	0.647
X62	3.000	1.785	0.724	0.000	0.440	0.442	1.000	0.930
X65	4.000	1.366	0.560	0.000	0.268	0.269	1.000	0.273
AF35050	3.000	1.165	0.318	0.000	0.142	0.142	1.000	0.143
CP00361	2.000	1.190	0.297	0.000	0.160	0.161	1.000	0.167
CP5096	2.000	1.250	0.352	0.000	0.200	0.201	1.000	0.212
CEDC055	4.000	1.659	0.790	0.000	0.397	0.400	1.000	0.397
CEDC033	3.000	1.165	0.318	0.000	0.142	0.142	1.000	0.142
CEDG100	5.000	1.849	0.916	0.000	0.459	0.462	1.000	0.459
CEDG013	3.000	1.490	0.576	0.000	0.329	0.331	1.000	0.329
CEDG15	2.000	1.051	0.117	0.000	0.049	0.049	1.000	0.049
CEDG24	2.000	1.568	0.548	0.000	0.362	0.364	1.000	0.362
CEDG035	3.000	1.490	0.576	0.050	0.329	0.331	0.848	0.329
CEDG41	3.000	1.078	0.177	0.013	0.073	0.073	0.828	0.073
CEDG60	2.000	1.406	0.464	0.000	0.289	0.291	1.000	0.289
CEDG70	2.000	1.438	0.483	0.000	0.305	0.307	1.000	0.305
CEDG97	4.000	1.813	0.886	0.000	0.448	0.451	1.000	0.454
CEDG116	2.000	1.078	0.160	0.000	0.072	0.073	1.000	0.072
CEDG136	5.000	1.883	0.876	0.000	0.469	0.472	1.000	0.942
CEDG150	3.000	1.106	0.227	0.000	0.096	0.097	1.000	0.096
CEDG185	3.000	1.497	0.597	0.000	0.332	0.334	1.000	0.334
CEDG267	3.000	1.349	0.466	0.000	0.258	0.260	1.000	0.277
CEDG290	2.000	1.025	0.067	0.000	0.025	0.025	1.000	0.025
DMSSR080	3.000	1.967	0.856	0.000	0.492	0.495	1.000	0.947
DMSSR199	4.000	1.945	0.819	0.000	0.486	0.489	1.000	0.908
DMSSR043	3.000	1.316	0.442	0.000	0.240	0.242	1.000	0.240
DQ9393	2.000	1.406	0.464	0.000	0.289	0.291	1.000	0.289
DQ469293	2.000	1.503	0.517	0.000	0.335	0.337	1.000	0.335
VR022	3.000	1.349	0.466	0.000	0.258	0.260	1.000	0.258
J01263	4.000	1.397	0.572	0.000	0.284	0.286	1.000	0.284
MBSSR008	2.000	1.503	0.517	0.000	0.335	0.337	1.000	0.335
PvM22	2.000	1.568	0.548	0.000	0.362	0.364	1.000	0.362
VM27	2.000	1.133	0.234	0.000	0.117	0.118	1.000	0.117
VR023	2.000	1.025	0.067	0.000	0.025	0.025	1.000	0.025
VR032	2.000	1.311	0.400	0.000	0.237	0.239	1.000	0.237
BMD8	3.000	1.291	0.453	0.000	0.225	0.227	1.000	0.233
Mean	3.562	1.825	0.694	0.050	0.376	0.378	0.905	0.427

*Na, number of alleles; Ne, number of effective alleles; I, Shannon’s information index; Ho, observed heterozygosity; He, expected heterozygosity; uHe, unbiased expected heterozygosity; F, fixation index; PIC, polymorphic information content.*

### Cluster-Based Genetic Diversity

**FIGURE 1:**
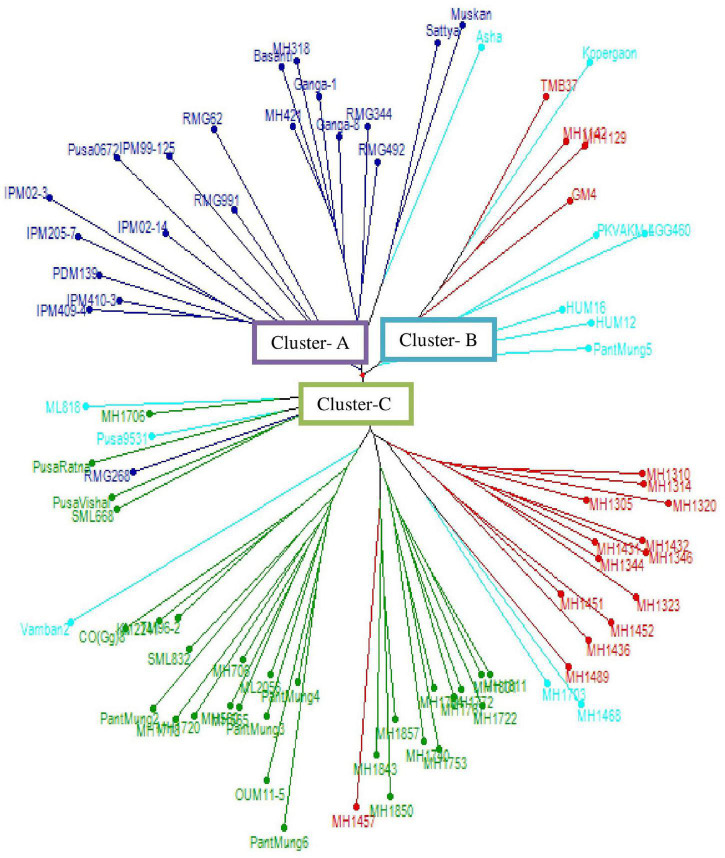
Dendrogram representing the genetic relationship among mung bean genotypes using weighted neighbor-joining (WNJ).

The WNJ analysis (Figure 1) distributed 80 genotypes into three major clusters (A–C). Among these clusters, cluster C was the biggest one accommodating 50 (62.5%) genotypes followed by cluster A with 20 (25%) genotypes and cluster B with 10 (12.5%) genotypes.

Cluster A could be further subgrouped into two subclusters, namely, AI and AII, both these subclusters consisted of released varieties only. Subcluster AI (10 varieties) had seven released varieties developed at the ICAR-Indian Institute of Pulses Research, Kanpur (ICAR-IIPR), two at Rajasthan Agriculture Research Institute (RARI), Durgapura, and one at ICAR-Indian Agricultural Research Institute (ICAR-IARI), New Delhi. Subcluster AII (10 varieties) consisted of six released varieties developed at CCS HAU, Hisar, two at RARI, Durgapura and two at Rajasthan Agriculture University, Regional Research Centre (RAU RRS), Ganganagar.

Cluster B accommodated nine released varieties and one advanced genotype which could be further grouped into three subclusters, namely, BI, BII, and BIII. Subcluster BI (five genotypes) accommodated four released varieties, i.e., one each from Bhabha Atomic Research Centre (BARC), Trombay; Anand Agricultural University (AAU), Anand; CCS HAU, Hisar, and Dr. Panjabrao Deshmukh Krishi Vidyapeeth (PDKV), Akola and one advanced genotype from CCS HAU, Hisar. Subcluster BII consisted of two released varieties, i.e., one each of PDKV, Akola and Agriculture Research Station, Lam (ARSL), Andhra Pradesh. Subcluster BIII comprised of three released varieties, two developed at Banaras Hindu University (BHU), Varanasi and one at Govind Ballabh Pant University of Agriculture and Technology (GBPUA&T), Pantnagar.

Cluster C (50 genotypes) could be further divided into five subclusters, from CI to CV. Subclusters CI and CII accommodated 15 and 12 advanced breeding lines from CCS HAU, Hisar, respectively. Subcluster CIII consisted of a total of 16 genotypes including 11 released varieties developed at different centers [1 each developed at Tamil Nadu Agricultural University-National Pulses Research Centre (TNAU-NPRC), Vamban; Tamil Nadu Agricultural University (TNAU), Coimbatore; Chandra Shekhar Azad University of Agriculture and Technology (CSAUAT), Kanpur; BARC, Trombay; Odisha University of Agriculture and Technology (OUAT), Berhampur; 2 developed at Punjab Agricultural University (PAU), Ludhiana; 4 at GBPUA&T, Pantnagar; and 5 advanced genotypes developed at CCS HAU, Hisar. Subcluster CIV (five genotypes) comprised three released varieties of ICAR-IARI, New Delhi and one each of RARI, Durgapura, and PAU, Ludhiana. Subcluster CV consisted of two genotypes, i.e., one advanced genotype of CCS HAU, Hisar and one released variety of PAU, Ludhiana (Table 3).

**TABLE 3:** Grouping of released and advanced breeding lines based on weighted neighbor-joining and population genetic structure.

Genotypes	Sub population/color code	WNJ clustering	Pedigree	Source
Asha	Admixture	AII	K 851 × L 24-2	CCS HAU, Hisar
HUM 12	Admixture	BIII	HUM 5 × DPM 90-1	BHU, Varanasi
HUM 16	Admixture	BIII	Pusa bold1 × HUM 8	BHU, Varanasi
LGG 460	Admixture	BII	Lam M2 × ML 267	ARS, Lam
MH 1468	Admixture	CI	MH 318 × AKM 9904	CCS HAU, Hisar
MH 1703	Admixture	CI	IPM 02-17 × MH 521	CCS HAU, Hisar
Kopergaon	Admixture	BI	CO 5-KM 2 × MG 50-10 (G)	Maharashtra
ML 818	Admixture	CV	5145/87 × ML 267	PAU, Ludhiana
Pant Mung 5	Admixture	BIII	Selection from VC 6368	GBPUA&T, Pantnagar
PKV AKM-4	Admixture	BII	BM 4 × PS 16	PDKV, Akola
Pusa 9531	Admixture	CIV	Selection from NM 9473	IARI, New Delhi
Vamban 2	Admixture	CIII	VGG 4 × MH 309	NPRC, Vamban
GM 4	Red (SP1)	BI	GM-3 × Pusa 9333	AAU, Anand
MH 1129	Red (SP1)	BI	Muskan × BDYR 2	CCS HAU, Hisar
MH 1142	Red (SP1)	BI	Muskan × BDYR 2	CCS HAU, Hisar
MH 1305	Red (SP1)	CI	MH 98-1 × MH 565	CCS HAU, Hisar
MH 1314	Red (SP1)	CI	MH 3-18 × Pusa 0672	CCS HAU, Hisar
MH 1315	Red (SP1)	CI	MH 3-18 × Pusa 0672	CCS HAU, Hisar
MH 1320	Red (SP1)	CI	MH 421-S-14-3	CCS HAU, Hisar
MH 1323	Red (SP1)	CI	MH 3-18 × AKM 99-4	CCS HAU, Hisar
MH 1344	Red (SP1)	CI	Muskan × BDYR 2	CCS HAU, Hisar
MH 1346	Red (SP1)	CI	Muskan × BDYR 2	CCS HAU, Hisar
MH 1431	Red (SP1)	CI	Muskan × BDYR 2	CCS HAU, Hisar
MH 1432	Red (SP1)	CI	Muskan × BDYR 2	CCS HAU, Hisar
MH 1436	Red (SP1)	CI	Muskan × BDYR 2	CCS HAU, Hisar
MH 1451	Red (SP1)	CI	MH 98-1 × Pusa 0672	CCS HAU, Hisar
MH 1452	Red (SP1)	CI	MH 98-1 × Pusa 0672	CCS HAU, Hisar
MH 1457	Red (SP1)	CII	MH 98-1 × MH 565	CCS HAU, Hisar
MH 1489	Red (SP1)	CI	MH 318 × Pusa 0871	CCS HAU, Hisar
TMB 37	Red (SP1)	BI	Kopergaon × TARM-2	BARC, Trombay
CO(Gg) 8	Blue (SP3)	CIII	COGG 923 × VC 6040	TNAU, Coimbatore
KM 2241	Blue (SP3)	CIII	Samrat × PDM 54	CSAUAT, Kanpur
MH 1706	Blue (SP3)	CV	IPM 02-17 × MH 565	CCS HAU, Hisar
MH 1718	Blue (SP3)	CIII	KM 2241 × MH 521	CCS HAU, Hisar
MH 1720	Blue (SP3)	CIII	IPM 02-19 × MH 565	CCS HAU, Hisar
MH 1722	Blue (SP3)	CII	Pusa 0672 × MH 521	CCS HAU, Hisar
MH 1740	Blue (SP3)	CII	IPM-409-4 × MH 318	CCS HAU, Hisar
MH 1753	Blue (SP3)	CII	MH 421 × IPM 205-7	CCS HAU, Hisar
MH 1754	Blue (SP3)	CII	MH 421 × IPM 205-7	CCS HAU, Hisar
MH 1767	Blue (SP3)	CII	MH 534 × MH 318	CCS HAU, Hisar
MH 1772	Blue (SP3)	CII	VGG-rt-1 × Sattya	CCS HAU, Hisar
MH 1801	Blue (SP3)	CII	IPM 02-17 × MH 521	CCS HAU, Hisar
MH 1811	Blue (SP3)	CII	Sattya × IPM 409-4	CCS HAU, Hisar
MH 1843	Blue (SP3)	CII	LGG 460 × Sattya	CCS HAU, Hisar
MH 1850	Blue (SP3)	CII	Sattya × IPM 409-4	CCS HAU, Hisar
MH 1857	Blue (SP3)	CII	Sattya × MH 318	CCS HAU, Hisar
MH 560	Blue (SP3)	CIII	Asha × BDYR 1	CCS HAU, Hisar
MH 565	Blue (SP3)	CIII	Asha × BDYR 1	CCS HAU, Hisar
MH 706	Blue (SP3)	CIII	MH 96-1 × BDYR 2	CCS HAU, Hisar
ML 2056	Blue (SP3)	CIII	ML 1165 × ML 1191	PAU, Ludhiana
OUM 11-5	Blue (SP3)	CIII	Mutant of Dhauli	OUAT, Berhampur
Pant Mung 2	Blue (SP3)	CIII	Mutant of ML-26	GBPUA&T, Pantnagar
Pant Mung 3	Blue (SP3)	CIII	LN 294-8 × L 80	GBPUA&T, Pantnagar
Pant Mung 4	Blue (SP3)	CIII	T 44 × UPU 2	GBPUA&T, Pantnagar
Pant Mung 6	Blue (SP3)	CIII	Pant M 2 × AMP 36	GBPUA&T, Pantnagar
Pusa Ratna	Blue (SP3)	CIV	VC 6368 × ML 267	IARI, New Delhi
Pusa Vishal	Blue (SP3)	CIV	Selection from NM 92	IARI, New Delhi
SML 668	Blue (SP3)	CIV	Selection from NM 94	PAU, Ludhiana
SML 832	Blue (SP3)	CIII	SML 302 × Pusa bold1	PAU, Ludhiana
TM 96-2	Blue (SP3)	CIII	Kopergaon × TARM-2	BARC, Trombay
Basanti	Green (SP2)	AII	Asha × PDM 90-1	CCS HAU, Hisar
Ganga-1	Green (SP2)	AII	Local selection from Kaluwala	RAU RRS, Ganganagar
Ganga-8	Green (SP2)	AII	K 851 × Pusa 105	RAU RRS, Ganganagar
IPM 02-14	Green (SP2)	AI	IPM 99-125 × Pusa bold2	IIPR, Kanpur
IPM 02-3	Green (SP2)	AI	IPM 99-125 × Pusa bold2	IIPR, Kanpur
IPM 205-7	Green (SP2)	AI	IPM 02-1 × EC 398889	IIPR, Kanpur
IPM 409-4	Green (SP2)	AI	PDM 288 × IPM 03-1	IIPR, Kanpur
IPM 410-3	Green (SP2)	AI	IPM 03-1 × NM 1	IIPR, Kanpur
IPM 99-125	Green (SP2)	AI	PM 3 × APM 36	IIPR, Kanpur
MH 318	Green (SP2)	AII	Asha × BDYR 1	CCS HAU, Hisar
MH 421	Green (SP2)	AII	Muskan × BDYR 2	CCS HAU, Hisar
Muskan	Green (SP2)	AII	PDM 116 × Gujarat-1	CCS HAU, Hisar
PDM 139	Green (SP2)	AI	ML 20/19 × ML 5	IIPR, Kanpur
Pusa 0672	Green (SP2)	AI	11/395 × ML 267	IARI, New Delhi
RMG 268	Green (SP2)	CIV	R 288-8 × J 781	RARI, Durgapura
RMG 344	Green (SP2)	AII	Mung selection-1 × J-45	RARI, Durgapura
RMG 492	Green (SP2)	AII	Mutant of RMG 62	RARI, Durgapura
RMG 62	Green (SP2)	AI	R 288-8 × China mung	RARI, Durgapura
RMG 991	Green (SP2)	AI	RMG 268 × UPM 98	RARI, Durgapura
Sattya	Green (SP2)	AII	PDM 116 × Gujarat-1	CCS HAU, Hisar

### Population Genetic Structure

**FIGURE 2:**
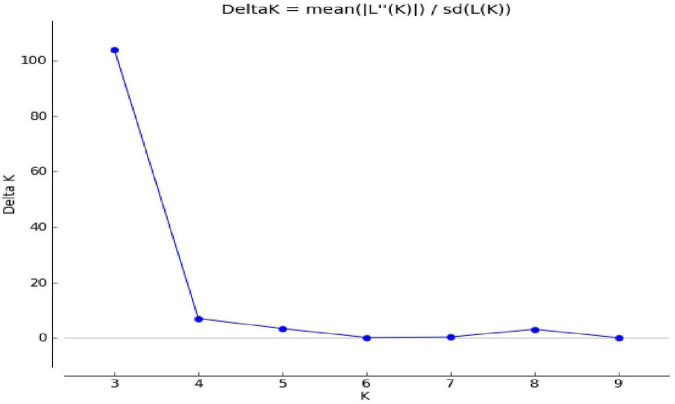
Population estimation using LnP (D) derived Δ*k* (*K* = 2–10).

**FIGURE 3:**
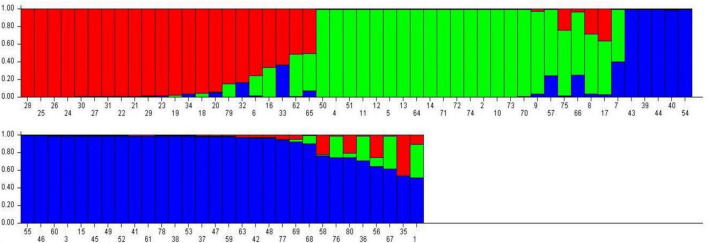
Bar graph representing population genetic structure of mung bean genotypes performed by admixture method in STRUCTURE.

Population genetic structure was used to analyze the structure of the population in the context of genetic diversity and the relatedness of the individuals within the group. Delta *K* value was used to estimate the significant number of SPs in all genotypes at the molecular level (Figure 2) by Evanno table. Population structure categorized the 80 cultivars and advanced mung bean genotypes into three genetically distinct SPs, namely, SP1 (marked by red), SP2 (green), and SP3 (blue) along with admixture group SP4 (mixture of colors) (Figure 3). Genotypes with *Q* values ≥0.7 were considered pure, while genotypes having <0.7 scores were considered admixture. Out of 80 genotypes, 68 (85%) resembled their hierarchy, and 12 (15%) were observed as the admixture form. The maximum number of genotypes (30) were grouped in SP3, followed by SP2 (20), SP1 (18), and SP4 (12).

The 18 genotypes in SP1 consisted of one released variety each developed by AAU, Anand, CCS HAU, Hisar, and BARC and fifteen advanced genotypes developed at CCS HAU, Hisar. SP2 accommodated 20 (25%) released varieties developed at CCS HAU, Hisar (5); RAU RRS, Ganganagar (2); RARI, Durgapura (5); ICAR-IIPR, Kanpur (7); and ICAR-IARI, New Delhi (1). SP3 comprised of 30 (37.5%) genotypes which included one variety each of TNAU, Coimbatore; OUAT, Berhampur; BARC, Trombay; and CSAUAT, Kanpur; 3 of PAU, Ludhiana; 4 of GBPUA&T, Pantnagar; 2 of ICAR-IARI, New Delhi; and 17 advanced genotypes developed at CCS HAU, Hisar. SP4 consisted of 10 released varieties and two advanced genotypes (15%). One released variety each belonged to CCS HAU, Hisar; GBPUA&T, Pantnagar; PAU, Ludhiana; ICAR-IARI, New Delhi; TNAU-NPRC, Vamban; and ARSL, Andhra Pradesh and two varieties each of BHU, Varanasi, and PDKV, Akola and the two advanced genotypes from CCS HAU, Hisar (Table 3).

### Genetic Diversity Within Subpopulations

**FIGURE 4:**
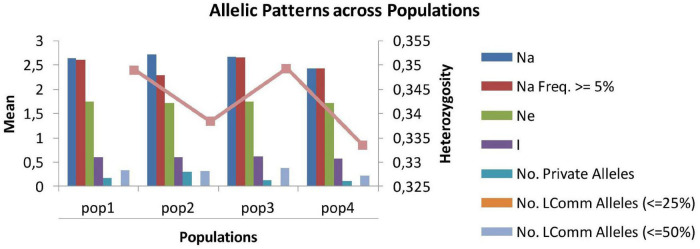
Graphical presentation of allelic patterns across the subpopulation.

The number of alleles per locus ranged from 2.43 (SP4) to 2.72 (SP2), and the number of effective alleles varied from 2.29 (SP2) to 2.66 (SP3) per locus. Shannon’s index minimum mean value was observed for SP4 (0.57) and maximum for SP3 (0.61), and the number of private alleles varied from 0.11 (SP4) to 0.30 (SP2). The mean value of expected heterozygosity ranged from 0.33 (SP4) to 0.35 (SP1 and SP3), and unbiased expected heterozygosity was slightly higher (0.36) for SP1 and SP3 and minimum for SP2 (0.34) (Table 4). For better visualization, genetic diversity within SPs is represented graphically (Figure 4). The genetic differentiation indices among the population (Fst) ranged from 0.001 (between SP1 and SP2, SP1 and SP3, SP2 and SP3, and SP2 and SP4) to 0.008 (between SP3 and SP4) (Table 5). The pair-wise Nei genetic identity value varied from 0.90 (SP1 vs. SP2) to 0.95 (SP1 vs. SP3) (Table 6). The differences within and among the groups studied from AMOVA analysis revealed that 7% of molecular variance was present among four SPs, 80% among individuals, and 13% of the total variation was observed within individuals (Table 7).

**TABLE 4:** Genetic diversity and mean allelic pattern across subpopulations of mung bean genotypes.

Population	Sub-population 1		Sub-population 2		Sub-population 3		Sub-population 4	
	Mean	SE	Mean	SE	Mean	SE	Mean	SE
Na	2.640	0.114	2.719	0.114	2.674	0.109	2.427	0.115
Na frequency ≥ 5%	2.607	0.114	2.292	0.097	2.663	0.109	2.427	0.115
Ne	1.744	0.072	1.709	0.072	1.741	0.075	1.712	0.072
*I*	0.604	0.041	0.595	0.039	0.612	0.039	0.573	0.043
No. private alleles	0.169	0.043	0.303	0.065	0.124	0.035	0.112	0.034
No. LComm alleles (≤25%)	0.000	0.000	0.000	0.000	0.000	0.000	0.000	0.000
No. LComm alleles (≤50%)	0.326	0.055	0.315	0.052	0.382	0.059	0.213	0.047
He	0.349	0.023	0.338	0.022	0.349	0.022	0.334	0.024
uHe	0.358	0.024	0.344	0.022	0.358	0.022	0.348	0.025

*Na, no. of different alleles per locus; Ne, no. of effective alleles per locus; I, Shannon’s index; He, expected heterozygosity; uHe, unbiased expected heterozygosity.*

**TABLE 5:** Pairwise population *F*st values.

Population	SP1	SP2	SP3	SP4
SP1	0.000	0.001	0.001	0.006
SP2		0.000	0.001	0.001
SP3			0.000	0.008
SP4				0.000

**TABLE 6:** Pair-wise population matrix of Nei genetic identity.

Population	SP1	SP2	SP3	SP4
SP1	1.000			
SP2	0.902	1.000		
SP3	0.952	0.944	1.000	
SP4	0.949	0.913	0.948	1.000

**TABLE 7:** Analysis of molecular variance.

Source	df	SS	MS	Est. Var.	%
Among population	3	232.160	77.387	1.228	7
Among individual	76	2262.053	29.764	13.760	80
Within individual	80	179.500	2.244	2.244	13
Total	159	2673.713		17.232	100

*df, degree of freedom; SS, sum of square; MS, mean sum of square; Est. Var., estimated variance; %, percentage of variance.*

### Linkage Disequilibrium

**FIGURE 5:**
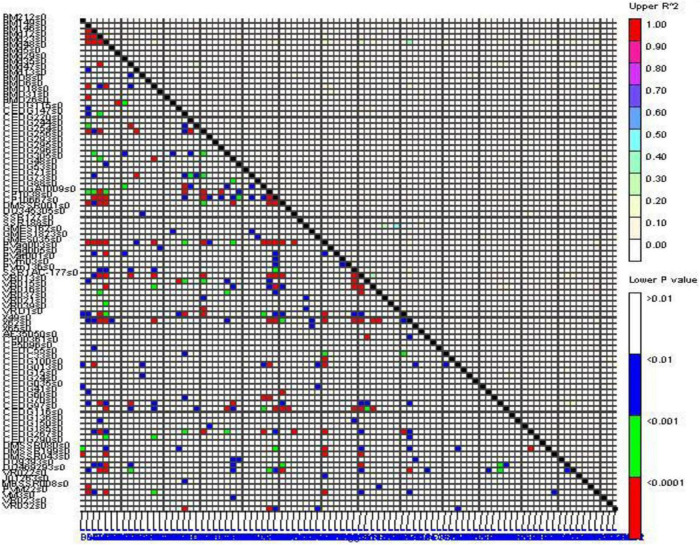
Triangle heat plot showing pairwise locus combination in mung bean genotypes.

Significant linkage disequilibrium (LD) blocks were observed in the genome-wide LD analysis as demonstrated by triangle heat plots for pair-wise LD between SSR using TASSEL software (Figure 5). The *R*^2^ value between marker pairs ranged from 0.1 to 0.49 (between VR039 and SSR188). The *R*^2^ value above 0.1 between marker pairs was considered to be in LD, and there were 75 marker pairs having the *R*^2^ value above 0.1. The marker BMd23 had the highest LD with 16 markers (i.e., BMd35, BM212, BMD6, CEDC55, CEDG185, CEDG70, CP1038, DMSSR199, DQ469293, DQ9393, GMES035, PVag005, PVM22, SSR1AC-177, VR015, and X49) followed by DQ469293 which had LD with 8 markers (i.e., X49, SSR1AC-177, DQ9393, CP10667, CEDG70, CEDG41, BMd35, and BMd23).

### Association Analysis

**FIGURE 6:**
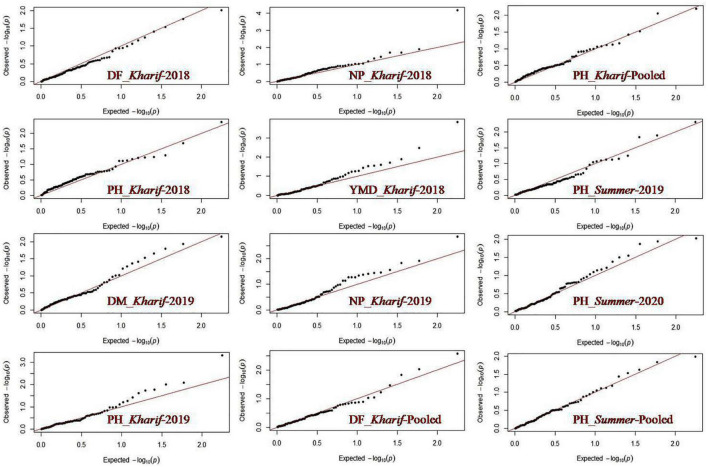
QQ plot showing association of markers with yield-related traits in mung bean.

Marker-trait association study was conducted using the mean values of all the SY-related traits based on a phenotypic evaluation over four environments and the allelic data of 89 polymorphic SSRs. A total of 38 marker-trait associations were observed to be significant for yield-related traits and YMD resistance by the generalized linear model (GLM-Q) at a corrected *p*-value of ≤0.0005 (Benjamini and Hochberg, 1995; Supplementary Table 5). Eleven marker-trait associations were found significant using the most accepted maximum likelihood model (MLM-Q + K) for four yield-related traits, namely, DF, DM, PH, and NP, and also for YMD resistance at *p*-value ≤0.01 (Table 8). This association explained high phenotypic variation, i.e., 41.55% through GLM and 13.57% through MLM. The maximum number of markers exhibited association with PH (five, i.e., DMBSSR043, CEDG97, DQ9393, CEDG295, and CEDG88) followed by YMD (two, i.e., J01263 and CEDG220). One MTA each for DF (VR022), DM (BM146), and NP (BMd12) in different seasons was identified from the MLM approach. In both GLM and MLM approaches, a total of four MTAs were found to be common across seasons for NB associated with BMd12, PH with DMBSSR043 and CEDG97, and DM associated with BM146. The marker BMd12 associated with NB expressed consistently in *kharif* (monsoon) 2018 and 2019. Similarly, CEDG88 associated with PH was identified consistently in the summer seasons during both years. CEDG97 and DQ9393 associated with PH were identified in *kharif* 2019 and pooled over *kharif* data. VR022 associated with DF was consistently identified in *Kharif* 2018, pooled data of *kharif* as well as the pooled data of *kharif* and summer (Figure 6). The MTA study also revealed the presence of pleiotropic markers in mung bean, i.e., a single marker associated with more than one trait, such as BMd12 associated with NP, PL, SW, and YMD, and CEDG97 associated with PL, PH, and NS. Likewise, the markers DMBSSR001, CP1038, VR021, BMd35, CP5096, BM146, DQ9393, and CEDG220 were also associated with different traits.

**TABLE 8:** Significant marker-trait associations identified from MLM (Q + K) approach in different environments.

*Kharif*-2018					*Kharif*-2019					*Kharif*-pooled				
Trait	Locus	Allele	*p*-Value	*R* ^2^	Trait	Locus	Allele	*p*-Value	*R* ^2^	Trait	Locus	Allele	*p*-Value	*R* ^2^
DF	VR022	175	0.0098	3.18	DM	BM146	285	0.0071	13.4	DF	VR022	175	0.0096	2.7
NP	BMd12	180	6.6E−05	2.95	NP	BMd12	195	0.0014	2.79	PH	CEDG97	110	0.0063	6.5
PH	DMSSR043	200	0.0043	13.6	PH	CEDG97	110	0.00049	10.3	PH	DQ9393	210	0.0088	2.9
YMD	J01263	180	0.00015	4.15	PH	DQ9393	210	0.0082	3.41	Summer-pooled				
YMD	CEDG220	170	0.0034	4.58	PH	CEDG295	190	0.01	3.4	PH	CEDG88	160	0.01	7.9

**Summer-2019**					**Summer-2020**					***Kharif*-summer-pooled**				

PH	CEDG88	160	0.0049	6.61	PH	CEDG88	160	0.0095	9.53	DF	VR022	175	0.01	2.7

*MLM, mixed linear model.*

## Discussion

Despite many research efforts undertaken for mung bean genetic improvement during the last few decades, its productivity still falls short of acceptable levels. The major reasons for stagnancy in its productivity are insufficient genetic variability, poor harvest index, high influence of GXE interaction, and susceptibility of many of the available cultivars to various biotic and abiotic stresses (Nair et al., 2019; Pratap et al., 2021), which ultimately result in yield instability. In addition, genetic improvement through breeding efforts is slow due to inadequate utilization of genomic resources and a dearth of trait-linked molecular markers to undertake molecular breeding for accelerated crop improvement. Molecular markers, owing to their environmental independence, are important tools to estimate the genetic variation present in the germplasm. These also have an advantage in the breeding program as these can be used to adjudge the presence or absence of a particular gene/allele or genomic segments contributing to the trait expression. Therefore, this study was conducted with a panel of 80 released varieties and advanced breeding lines of mung bean for the purpose of estimating the genetic diversity using molecular markers and detecting loci associated with yield attributing traits and YMD resistance by association analysis. At the phenotypic level, a considerable amount of variability was observed among the mung bean genotypes for all the studied characters. Furthermore, very less difference between PCV and GCV estimates was observed which indicated the inherent nature of variability and lesser influence of environmental factors on the expression of these traits.

Selection for yield may be effective if all the traits that directly or indirectly affect the yield are considered during selection. In-depth prior knowledge of the magnitude and direction of the association among the characters is imperative for operating an efficient selection program in crop plants. In the present investigation, SY was found positively associated with PL, NB, NP, and NS and negatively associated with YMD. The present results with respect to yield attributes are in consonance with the findings of Saeed et al. (2007), Win et al. (2009), Kumar et al. (2010), Khajudparn and Tantasawat (2011), Zaid et al. (2012), and Baisakh et al. (2016). A negative correlation between yield and YMD resistance was also reported by Alam et al. (2014) and Anuradha et al. (2019).

Initially, 164 SSRs from different *Vigna* species (adzuki bean, cowpea, mung bean, and common bean) were selected (Pratap et al., 2015; Suman et al., 2019; Singh et al., 2020). SSRs from related species, namely, cowpea (Li et al., 2001), common bean (Blair et al., 2013), and adzuki bean (Wang et al., 2004) could be easily cross transferred to mung bean in earlier studies. In this study also, most of the primer pairs from related species were amplified with varying allele sizes ranging between 100 and 310 bp and, therefore, exhibited their potential across closely related *Vigna* species (Pratap et al., 2015). All polymorphic primer pairs generated 317 polymorphic alleles with an average of 3.56 alleles per SSR locus, and the number of alleles at each locus (Na) varied from 2 to 7 which is consistent with earlier studies (Sangiri et al., 2007; Shrivastava et al., 2014; Singh et al., 2020). Heterozygosity was observed in nine SSR loci which ranged from 0.01 to 0.97 with an average of 0.05. The expected heterozygosity ranged from 0.02 to 0.78 with an average of 0.38. Therefore, this study suggests the existence of ample genetic diversity among the released and advanced mung bean genotypes used, and this may be useful for the selection of genotypes for hybridization programs directed toward mung bean improvement. The genotypes found highly diverse at the molecular level are expected to exhibit more heterotic effects in F_1_ generation, and the information generated in this study could be considered valuable for developing heterotic pool in mung bean.

The wide range of PIC values of SSRs indicated that the markers used in this study were ample to explore the genetic diversity among studied genotypes. The PIC value obtained in this study using *Vigna*-species-specific SSRs is in accordance with earlier studies (Tangphatsornruang et al., 2009; Lestari et al., 2014; Shrivastava et al., 2014; Pratap et al., 2015; Markam et al., 2018; Suman et al., 2019; Singh et al., 2020). Pratap et al. (2015, 2021) recorded maximum PIC value for J01263, VR0163, VR0338, and SSR-IAC-177 (0.89) followed by BMD-12 (0.88), and in this study also, the BMD-12 marker locus revealed high genetic variation (PIC 0.73) among different mung bean varieties. A total of 30 primers were observed to have a PIC value of ≥0.5 and 32 primers having above-average PIC value suggesting that the highly polymorphic SSRs would be a valuable resource for assessing the mung bean genetic diversity and QTL mapping studies.

The WNJ analysis distributed 80 genotypes into three major clusters (A, B, and C). Among these, cluster C was the major cluster comprising all the advanced genotypes while the other two clusters consisted of all released varieties developed at different institutes. The subclusters AI and AII consisted of released varieties only. In an earlier study, Suman et al. (2019) assessed the genetic diversity of 18 mung bean genotypes, and the dendrogram based on SSR data grouped the mung bean cultivars IPM 02-14 and PDM 139 (developed at ICAR-IIPR, Kanpur) in the same cluster and HUM 12, HUM 1, and HUM 16 (developed at BHU, Varanasi) and few other varieties in another similar cluster. Lestari et al. (2014), Chen et al. (2015), Pratap et al. (2016), and Kaur et al. (2018) also reported similar clustering results in mung bean. Most recently, Pratap et al. (2021) in their analysis of 41 released varieties and elite lines of mung bean also reported grouping of all the varieties developed at IIPR after the year 2000 in a single cluster.

Population genetic structure categorized all 80 mung bean genotypes into three genetically distinct SPs along with the admixture class as observed in WNJ analysis. Pratap et al. (2021) also grouped 41 mung bean elite lines in 3 SPs. Noble et al. (2018) also determined four SPs in the cultivated mung bean germplasm genotyped with integrated DArT and genotyping-by-sequencing (GBS) methodology. Qin et al. (2017) studied 338 genotypes of cowpea from different geographic regions of the world and found 3 SPs. Reddy et al. (2020) and Singh et al. (2020) also employed released varieties, advanced breeding lines, and exotic genotypes of mung bean and reported that the released varieties grouped together in one SP as also identified in this study. It is noteworthy that in the present investigation, both the cluster analysis and the population genetic structure categorized the genotypes in a similar manner as all advanced breeding lines were grouped into a single cluster or SP, while the released varieties developed at different institutes were categorized in different clusters or SPs. This study suggests that all the advanced genotypes and released varieties of mung bean from different institutes might have a certain degree of common ancestral history; therefore, population genetic structure was in agreement with genetic diversity analysis.

Association mapping is a powerful tool used for dissecting complex traits based on LD. It exploits historical and evolutionary recombination present in an unstructured population to map QTLs in higher resolution (Flint-Garcia et al., 2003). A significant and true marker-trait association can be utilized for MAS to improve breeding efficiency in terms of time and cost (Pratap et al., 2017; Singh et al., 2020). Significant LD blocks were observed in the genome-wide LD analysis with 80 SSR genotypic data, and a similar pattern of LD in different *Vigna* species was reported (Galeano et al., 2012; Xu et al., 2012; Noble et al., 2018; Kumar et al., 2019; Reddy et al., 2020). Ten significant marker-trait associations for yield-related traits and YMD resistance were identified over the four different environments along with their pooled data using the most accepted maximum likelihood model. However, few associations were consistently expressed across seasons. Singh et al. (2018) reported five molecular markers (CEDG044, CEDG256, cp05325, GMES0214, and VrD1) to be associated with 100-SW, three (CEDG166, VrD1, and MBSSR238) with the NP/plant, and two markers (CEDG056 and GMES0214) with the NS/pod in mung bean following QTL mapping based on single marker analysis in a recombinant inbred line (RIL) mapping population developed from the cross between MYMIV susceptible cultivar Sonali and resistant wild relative of mung bean (*V. radiata* var. *sublobota*). In our study, six markers, namely, VrD1, CEDG044, cp05325, GMES0214, CEDG166, and CEDG056 reported by Singh et al. (2018) as associated with different traits, had been employed but none of them could be found associated with any of the studied traits. This disagreement could be primarily due to the difference in the mapping population and the approach followed in the earlier study. Furthermore, the single-marker analysis used in the earlier study is not considered a robust approach to map quantitative traits and many times results in the spurious association. The limited number of recombination events in the biparental mapping population results in mapping QTLs in larger genomic intervals than the association mapping. These QTL flanking markers might not be associated with traits when employed in association mapping due to the existence of high recombination events which might break the linkage between earlier-associated markers with traits.

A number of earlier studies claim that YMD resistance in mung bean and other *Vigna* crops is governed by one or two quantitative genes. However, a few recent QTL and association mapping studies indicate that resistance is governed by multiple genes (Singh et al., 2018, 2020). In this study, five MTAs (BM146, BMd12, BMD26, CP1038, and CP5096) from GLM and two MTAs (CEDG220 and J01263) from the MLM approach were identified for YMD resistance. Singh et al. (2020) reported 14 and 12 MTAs linked with MYMIV resistance following GLM and MLM methods, respectively. Among these, the marker CP1038 common in both the studies was also identified in *kharif* 2019 and pooled over *kharif* data in this study. Besides the association of CP1038 with YMD, its association with PH and SY was also observed, and therefore, this genomic segment is considered to be pleiotropic. Furthermore, BM212 shown to be associated with MYMIV resistance by Singh et al. (2020) has a trait association with DM in our study. Singh et al. (2018) reported four QTLs linked with MYMIV resistance based on single marker analysis in a RIL mapping population developed from susceptible cultivar Sonali and resistant wild relative of mung bean (*V. radiata* var. *sublobota*) but none of them were found to be associated with YMD resistance in this study.

Few studies on mapping quantitative traits in mung bean following the association mapping approach have been reported till date in traits such as seed coat color (Noble et al., 2018), seed mineral content (Wu et al., 2020), MYMIV resistance (Singh et al., 2020), salinity tolerance (Breria et al., 2020), and phosphorus use efficiency (Reddy et al., 2020; Supplementary Table 6). However, this is the first report identifying MTAs for yield-related traits along with YMD resistance in mung bean. Nonetheless, a comparatively less number of MTAs was identified in this study which could be due to less number of markers deployed, and therefore, this warrants examining more markers, especially the mung bean-specific markers which have been developed in the last 3–4 years. This study not only identifies MTAs for various yield attributing traits but also validates the marker associated with YMD resistance identified in earlier studies. Therefore, this study would help in fine mapping of common YMD resistance loci identified across different studies and would eventually help in improving mung bean varieties for YMD resistance following fast track and precise molecular breeding with linked markers. Furthermore, the markers for yield-related traits would also be helpful in fast-track breeding for mung bean improvement utilizing these after validation across different populations.

## Conclusion

The population genetic structure analyses grouped the 80 mung bean genotypes into three major clusters and three genetically distinct SPs with one admixture SP based on 89 genome-wide polymorphic SSRs. This generated 317 polymorphic alleles with an average of 3.56 alleles per SSR locus. Both, i.e., cluster analysis and genetic population structure, categorized the advanced mung bean breeding genotypes in a single group/SP and the released varieties in other groups/SPs suggesting that the studied genotypes may have common ancestral history at some level. The genetic population structure was in agreement with the genetic diversity analysis. A total of 38 and 10 marker-trait associations for yield-related traits and YMD resistance by GLM and MLM methods, respectively, were identified as significant, and one SSR marker CP1038 associated with YMD resistance was validated. These associations may be useful in marker-assisted mung bean improvement programs in future after validation of the markers in biparental mapping populations.

## Data Availability Statement

The datasets presented in this study can be found in online repositories. The names of the repository/repositories and accession number(s) can be found in the article/Supplementary Material.

## Author Contributions

VR, RY, RS, and AP designed the experiment and wrote the manuscript. VR, APn, and GK conducted field and laboratory experiments and generated the data. VR, RY, PSS, and AP analyzed the data and interpreted the results. RY, AP, and RS supervised the research. RY, AP, PSS, and RS reviewed the manuscript. All authors read the manuscript and approved the submitted manuscript.

## Conflict of Interest

The authors declare that the research was conducted in the absence of any commercial or financial relationships that could be construed as a potential conflict of interest.

## Publisher’s Note

All claims expressed in this article are solely those of the authors and do not necessarily represent those of their affiliated organizations, or those of the publisher, the editors and the reviewers. Any product that may be evaluated in this article, or claim that may be made by its manufacturer, is not guaranteed or endorsed by the publisher.

## Footnotes

1
https://www.indostat.org

2
http://darwin.cirad.fr/
