# Supplementary material for: Association Mapping for Yield Attributing Traits and Yellow Mosaic Disease Resistance in Mung Bean [Vigna radiata (L.) Wilczek]
Source: Front Plant Sci. 2022 Jan 17;12:749439. doi: 10.3389/fpls.2021.749439 (PMC8801447; doi:10.3389/fpls.2021.749439)
Supplement: Supplementary file 1 [file Table_1.pdf]

Supplementary Table 1 Scoring of yellow mosaic disease in mungbean

Disease score	Disease reaction	Remarks
0	HR	No visible disease symptoms on leaves
1	R	< 5.0% disease symptoms on leaves
3	MR	5.1–15.0% disease symptoms on leaves
5	MS	15.1–30.0% disease symptoms on leaves
7	S	30.1–75.0% disease symptoms on leaves
9	HS	> 75.1% disease symptoms on leaves and sometimes may be on pods

HR - Highly resistant, R - resistant, MR - moderately resistant, MS - moderately susceptible, S - susceptible, HS - highly susceptible
