# Supplementary material for: Association Mapping for Yield Attributing Traits and Yellow Mosaic Disease Resistance in Mung Bean [Vigna radiata (L.) Wilczek]
Source: Front Plant Sci. 2022 Jan 17;12:749439. doi: 10.3389/fpls.2021.749439 (PMC8801447; doi:10.3389/fpls.2021.749439)
Supplement: Supplementary file 2 [file Table_2.pdf]

Supplementary Table 2 List of SSRs used in present investigation

Primers Name	Forward sequence	Reverse sequence
AF350505	CAGACATGCAAATTGGAAC	GGAGCACCAAAGATCATAGA
BM 146	GAGATGAGTCCTTTCCCTACCC	TGCAGACACAATTTATGAAGGC
BM 170	AGCCAGGTGCAAGACCTTAG	AGATAGGGAGCTGGTGGTAGC
BM 212	AGGAAGGGATCCAAAGTCACTC	TGAACTTTCAGGTATTGATGAATGAAG
BM149	CGATGGATGGATGGTTGCAG	GGGCCGACAAGTTACATCAAATTC
BMD-12	CATCAACAAGGACAGCCTCA	GCAGCTGGCGGGTAAAACAG
BMD-13	TCATGCCTGAGAAAGGGTCT	CCCTGCATCAGAAGTCCAAT
BMD-18	AAAGTTGGACGCACTGTGATT	TCGTGAGGTAGGAGTTTGGTG
BMd-2	AGCGACAGCAAGAGAACCTC	CAACAAACGGTGATTGACCA
BMD-23	GGCTTGGTCCTCTCATTGAA	TGGAAATTACCACCATGCAA
BMD-26	CTTGCCTTGTGCTTCCTTCT	TCCATTCCCAACCAAGTTTC
BMD-29	CTTCACCGATCTGACAGCAG	TTTCTCCACTGGAACACTCG
BMD-31	TGAAGAGGATCGCAAGGTTC	AGCCGAAACACTGTCCTTGT
BMD-35	TCTCTTCCTTACCCTGTTCTGC	GCGTGGACTTGAATGGTTTC
BMD-47	ACCTGGTCCCTCAAACCAAT	CAATGGAGCACCAAAGATCA
BMD-48	CCCCACCAACTCTTTCTTCC	CAGAATTGACTTGGCGAGAA
BMD-5	CGTGGACTTGAATGGTTTCAG	TCCTTACCCTGTTCTGCTTCTC
BMD-6	CATCGAATGCCCAAGAGAATA	CTCACTGTCTTCCATCCAAGC
BMD-8	TTCATCCTCTCTCCCGAACTT	CTTTTGTGGCTGAGACATGGT
CDEG 220	GGTATTGAAGTCACATGGTCC	GGTTGTTATCTTTGTGCACTCC
CDEG 244	GCATATAAGAAAAGCTTATCC	CTCTTGGAGTGATTTGATC
CEDC 033	GTGAGGTAGCTATGTAGCAC	ACTGGACCGACAAGAGTAAG
CEDC 055	CAAACACTTTTGTAACTCCC	GCTTCTAACCTTGATCCTTC
CEDC 139	CAAACTTCCGATCGAAAGCGCTTG	GTTTCTCCTCAATCTCAAGCTCCG
CEDC 302	GCATTGATGGAAGTAGTCAGTCG	CACCCTCTACATCACCGGTTG
CEDC 302	GCATTGATGGAAGTAGTCAGTCG	CACCCTCTACATCACCGGTTG
CEDG 003	CCACTTTCTCTTGACTTTGC	GACCAAAGTGAAGCCAAGAG
CEDG 003	CCACTTTCTCTTGACTTTGC	GACCAAAGTGAAGCCAAGAG
CEDG 008	GCCCATATTTTTACGCCCAC	GCCCATATTTTTACGCCCAC
CEDG 013	CGTTCGAGTTTCTTCGATCG	ACCATCCATCCATTCGCATC
CEDG 015	CCCGATGAACGCTAATGCTG	CGCCAAAGGAAACGCAGAAC
CEDG 024	CATCTTCCTCACCTGCATTC	TTTGGTGAAGATGACAGCCC
CEDG 030	TGAGGGAATGGGAGAGAGGC	TCCGCAGATAGAGGCTCACG
CEDG 035	TGGTTGGATGAAAGCGTGTG	CTGTGAGAGGTTCAACAACC
CEDG 036	CAGGTATTGTGCAGAGAGAC	TGCACCCAAAAGCTGTAAGC
CEDG 041	GCTGCATCTCTATTCTCTGG	GCCAACTAGCCTAATCAG
CEDG 044	TCAGCAACCTTGCATTGCAG	TTTCCCGTCACTCTTCTAGG
CEDG 048	TCTCTTCCTCTATGGCTTGG	GCTCCTCTTTTTGCTGCATC
CEDG 050	GGCAGAATCGTACAAGTG	GTCAGATTCTCGCTTGCATG
CEDG 053	CGTTGCAGAGCGGTGGTGG	GCCTTTGCTTCCCATCCATG
CEDG 064	TGTAAGGTCACTTTGGCCTCAAG	TTAAGTTGACTCGTTGCCCTTTG
CEDG 070	CCGATCAAACTCTCCATGCTCG	TAATTTCATTGCTTTTCCCTCC
CEDG 071	GGTCCATTGAGACGGATCGAG	TCCCACCTCAGCGGAATCC
CEDG 073	CCCCGAAATTCCCCTACAC	AACACCCGCCTCTTTCTCC
CEDG 084	ATCAACTGAGGAGCATCATCGA	CAACATTTCAACCTTGGGACAG
CEDG 088	TCTTGTCATTTAGCACTTAGCACG	TTGTTGTTTACTAAGAGCCCGTGT
CEDG 096A	TTACGAAACTGTGGCCTTCAT	TGAACAAAGATGACTTCGGTG
CEDG 100	CCCATCAAGTAACTACATAACA	ATGTGGGACTGGACAAATAAAA
CEDG 113	CGTTCGAGTTTCTTCGATCG	ACCATCCATCCATTCGCATC
CEDG 115	GGCTCATTGTACCACTGGATAT	ATGCCTCCTTTCAGGTGATTGT
CEDG 116	TTGTATCGAAACGACGACGCAGAT	AACATCAACTCCAGTCTCACCAAA
CEDG 118	AACCCAACCAACCCTTGTGGTAAG	GCTGGAATCATAATACCGCCTTGT
CEDG 121	CTTTCAAAATAATGTTGAGGCATA	CAATACATAAATAACCTTTTCTGC
CEDG 128	CTGCCAAAGATGGACAACTTGGAC	GCCAACCATCATCACAGTGC
CEDG 136	GTTCCAAGTCTCCAATCCGTAC	CACTTCACTAGAACTGGTTCAG
CEDG 146	GGTGATCGGATTTCAGAG	GGAGAAGAGAATAGAGACG
CEDG 147	CTCCGTCGAAGAATTGGTTGAC	GCAAAAATGTGGCGTTTGGTTGC
CEDG 150	GAAGGGAATGAAAATGAAACCC	GTTCAATCCATTCAGTCTCC
CEDG 159	CACATGTGACCAACAAGATTC	GAGTTAGTGATTGAACGAAGC
CEDG 166	GGTACAACATTCTTCTATTTG	GGCTTATGAGTTTATCTTATC
CEDG 168	CTGCTTGGTGTTGAAGCTTC	CATTCTACATTCCAGACCTGC

CEDG 185	CACGAACCGGTTACAGAGCG	CATCGCATTCCCTTCGCTGC
CEDG 191	CAATAAGCAATCTGTGGAGAG	CTGCAGGAAACTTGGAATTGC
CEDG 204	CCTTGGTTGGAGCAGCAGC	CACAGACACCCTCGCGATG
CEDG 211	GAGTGTGCATATGTGAGAG	CAAGTCTAATCTCTGACTCC
CEDG 218	CTTGCACCACAGTCTCCTTG	CCAATTGGAGGGTTGGTGTC
CEDG 225	GAGGAAGTGTTGCAGCACC	GTAGACTCTGCAGAGGGATG
CEDG 247	GTAGACACTGATCATCACC	GACCATCATCGATACGATTC
CEDG 254	CGATGTCTCTTGCTTCAAGG	GTGAAGGACTAGCCAAGTTTG
CEDG 271	GCACTAAAGTTAGACGTGGTTC	CACTCCCACTGCCAAACAAGG
CEDG 290	GACACTCTTTGTTTGTAGG	CAGTGATCACTCTGGTTG
CEDG 291	CCTCAAGTGGGGTTACC	GGTCAACCTCATTCTCCC
CEDG 293	GGATGGTAATGGTAGTTGCTG	CTTCTAGAAACCCGTCACTG
CEDG 295	CAAAGGTTAGATCCAACATCG	GGTTAGTCATCAACAACTCC
CEDG 296	CCAGCAGAGAAGCCAGATTAC	GCAGTGCAAGCATGGATGAAG
CEDG 297	GAACGCCATGGCACGAATGG	CGTTGTCGAACTTGTCGTACG
CEDG 305	GCAGCTTCACATGCATAGTAC	GAACTTAACTTGGGTTGTCTGC
CEDG012	AAAGCACTCGTAGTGGATGG	TAGCAACACCTAGGAGCAAC
CEDG020	TATCCATACCCAGCTCAAGG	GCCATACCAAGAAAGAGG
CEDG026	TCAGCAATCACTCATGTGGG	TGGGACAAACCTCATGGTTG
CEDG027	ACTTGGGGTTTGAGATGTGG	TCATTTTGGCCACTCAGTGC
CEDG056	TTCCATCTATAGGGGAAGGGAG	GCTATGATGGAAGAGGGCATGG
CEDG060	TGCATCGTCTCATGCGACCAG	ATTCTGGCAGCGGAGAAACC
CEDG075	GCGACCTCGAAAATGGTGGTTT	TCACCAACTCACTCGCTCACTG
CEDG097	GTAAGCCGCATCCATAATTCCA	TGCGAAAGAGCCGTTAGTAGAA
CEDG174	GAGGGATCTCCAAAGTTCAACGG	GAAGGCTCCGAAGTTGAAGGTTG
CEDG176	GGTAACACGGGTTCAGATGCC	CAAGGTGGAGGACAAGATCGG
CEDG186	GGATGGGAGAGTAAGAAG	GCATGGCATGATGACTTG
CEDG256	CCTTCACTATGTCCACATCC	GTTGTTGGTCGGTTCAGAG
CEDG267	GTGCTTGCATATGCAATGTCC	GGCATCTAAGAAATCCATGTCG
CEDGAT 009	GACTTAACACGTCTGAAAG	GAAGTTAGTGCAATCCAC
CP 00226	ACATTTGGAGGCTCAAGTTGGAAA	GGCCTATTACTACAGTCTGGAGGG
CP 08695	ATTAGTGGCGGCTCACAACAGTTC	ACGGCCTGTTGTCGGATAGAATAA
CP 10667	CCAAGGATTCATTCCTCACAAACC	TATTGGCCACTGAGAAACCCAACT
CP 1225	TCATCAGCTCATAAGCCACTGGTA	TGAGTATTTAACCTGCGACAGGCA
CP 5096	AATAGTTGCACAGCCCAAAAGGAA	CAGAAAACGGAGACGACTGTCTGA
CP00361	TAAATGCTAATACTCGCCGCCTTG	ACTGTGTCCGTTCGTCCTTTTCTC
CP00464	TGTAAGGTCACTTTGGCCTCAAG	TTAAGTTGACTCGTTGCCCTTTG
CP02662	CACTTCAGTTTCTTGCTCTCATAGGC	GCTCAAACTGCGAGACATTCAAGA
cp05325	GTCCAAGTGGAAGAAAGAAGGGGT	GGAGCTACCTTTTCAGTGTGGAGC
CP09781	CTGACGCATTCAGCATTTTACAGC	GGAAATACGGTTGCGTCCATGTAT
CP1038	TTTTGACAGAAGAAACGTGGTGGA	GGGGTATGTCTGAAAGTTCAACGC
DMB SSR001	GCAATCGATGGAGATTTGAA	TTCATGGATTGTTTTCCTTCC
DMB SSR008	AGGCGAGGTTTCGTTTCAAG	GCCCATATTTTTACGCCCAC
DMB SSR016	GTGCGAAAATTCGAAATGGT	TCAACTCAAGCAATGCTAAGTCA
DMB SSR024	TTTCTGCGAAGAATCTGAAGG	TTTTGAGTACCATGCTCTTCCT
DMB SSR035	TTCCACAAAGCTTGCTTCAGAT	TTGCATTCCAGCACATTTACA
DMB SSR151	AATGAAGGCTTGTCAAATCCA	TTATTTCACCTTGGCTGGATCA
DMB-SSR 059	TGCCAGATTTGAGAAGAAAGGT	CATGCATGTGGATAAGAATTCAG
DMB-SSR043	GATATGGTTTTAGAGGCGATCACA	CAATACTTCGCCAACAAATCA
DMB-SSR080	CGAGGCAGAGAAACCTTAAGAA	GCTCGATACTCTTGGGTTGAA
DMB-SSR199	AGAAATTAAATCCCCGTCTGCT	AGAGACAGAAGCTCTGGATGTTTT
DQ345305	GAGGCCAATCCCATAACTTT	AGCACCACATCAGAGATTCC
DQ469392	TCCCGATTTATAGTTCTCATTT	AGGGACCTCCTTCATCTC
DQ469393	CATTGAGATTTGAGGTTTCGTT	AGGTATTTCCATCGTGCTTTTC
GMES 0035	TGGTTGGATGAAAGCGTGTG	CTGTGAGAGGTTCAACAACC
GMES 0208	TTCTCTCGGAGCCGCTATTA	GGAACTCCAGGAGAGGGAAC
GMES 0211	ATATGTGGAGGTTCCGGTTG	GCCACAAGGCGTTAACAAAA
GMES 0216	CCGGGACAGGGTTTCTAACT	CCGAAGAAGACGACGAAATC
GMES 0337	TCCCTTTCTGCTTTTGCCTA	GAAATCCACAGCATGCTTCA
GMES 2320	GGAGATTCTGGTCCGTGAAA	GGATGTAACGAAAGCGAGGA
GMES0118	CAACCCTCTGTGTCTCGTCA	GAAGAAGAAGAAGAAGTGAAGGAAGA
GMES0162	TTTGGAGAACGGGAATTGAG	TATGAAAGCCCAGGAAATGC
GMES0214	GGGTTTCTTGGAAGGGTTTC	CTGCTTCCTCACCTGTCTCC

GMES1823	TGGACGACGAAACAGATCAA	ATCCAGCTGGTTTTCTGTGC
J01263	ATGCATGTTCCAACCACCTTCTC	GGAGTGGAACCCTTGCTCTCATC
JMES1424	TCTTCGGTGTTGCAATCAAG	ACAACCTTCAAACTGGCTGG
MB-SSR 008	ACCATTACCTCCACAATCTC	AATGGAGTTCTACGTGATGG
PV-ag003	TCACGTACGAGTTGAATCTCAGGT	GGTGTCGGAGAGGTTAAGGTTG
PV-ag005	GCTCACGTACGAGTTGAATCTCAG	ATCTGAGAGCAGCGACATGGTAG
PV-at001	GGGAGGGTAGGGAAGCAGTG	GCGAACCACGTTCATGAATGA
PvM 17	AGGATGGGTTCCGTGCTT	GGCAGCTTCCACATCGTC
PvM03	CCGCCTTCTTCTTCTTCTTC	CGGCGAGTCATCTTTTCC
PvM13b	GAGAAGCCGCAGAGAGGA	AGATGCCGCGAACAGAAC
PvM22	ACTCTCACAATGGCGGAATC	GGCGTTTTCTCCCTCTTCTT
SSR-IAC 127	GAGGCTAGCCCAACTTA	AGCGCAAGACTTTACTACTC
SSR-IAC 188	CCTGCCTTTGCCACTCCTC	CTCCTTCTACCCAGCCAAACC
SSR-IAC 195	TGGACATCAAACAAACAAAAA	TGCATCGGCAGTTCATCA
SSR-IAC-177	ACGGTTGGAGAAGATGATGA	ACCAATACAGGAAAGGGAGTT
VM 27	TCAACAACACCTAGGAGCCAA	ATCGTGACCTAGTGCCCACC
VM 37	TGTCCGCGTTCTATAAATCAGC	CGAGGATGAAGTAACAGATGATC
VR013	GCCCAGATTTGTTCATCCTAGA	ACTGTTTTGAGTGGGGAAAAGA
VR015	AAGATCACACACAACCAACCC	AATTAGTTCCACAGGCCAGATT
VR016	AGGAGAAATTGTTGTTGTTCGG	GTGTTGATTGTTAGGGAGGGAG
VR018	ATACAAGGGCAGGTGTAGCATC	CAGAAAACTTCATCCCCAGCTA
VR021	TTCCCTGTGTCCTTATATGTCC	GAGGATAGTGAATTTTGAAGGC
VR022	CAGCAACAGAACTACAATCCCA	CGGCAATCCTCCTATATTCATT
VR024	GCTCTAAAACACGAAAGGGGT	TCATGGTGGAAGAAAAGCAA
VR032	GATGGCTCTGCATTGAAACC	GATCTTCCCAACTTTCCCTCTC
VR033	ACTGAAGAGAATGGGTTAGGGG	TCACATTTGTTGGGTTGAAGAG
VR035	GCCCGATGTCCTAGCTTTTAG	CCTCAAAACAATCAGAACTCTCG
VR037	TCTCAGCATCTGTGGTGGTAGT	AGAATCCAACAACTCCTGCTTC
VR039	TGCTAAAGGTTTCCTCTCAACT	GAATGAAGTCACGCACACAA
VR045	TCTTTTCTATGTATGGCGCAAC	TTGGCTTTCGTATTTCCTCAGT
VR048	GGCAGGGAAGGAGGAAAA	CAGCCACAACAAGGCACA
VrD1	CAGCTTCTTGTTCTTGCTCC	CGAATGTGCACAGGTGGTGT
X 34	CGGAAGAAGAACGCAGAGTG	GCATCAACAAGGACTTCTGC
X39	CGATGTCTCTTGCTTCAAGG	GTGAAGGACTAGCCAAGTTTG
X40	GATTGGGAATCTGCTGTTG	GTGATCCACACACACAGTAC
X49	GGCAGAATCGTACAAGTG	GTCAGATTCTCGCTTGCATG
X56	GGTCCCAAAATCACCCAG	GGTTCATTTGGAGCACTGAG
X62	TGGGCTACCAACTTTTCCTC	TGAGCGACATCTTCAACACG
X65	CAACATTTCAACCTTGGGACAG	ATCAACTGAGGAGCATCATCGA
X87	GTCCTTGTTTTCCTCTCCATGG	CATCAGCTGTTCAACACCCTGTG
