# Supplementary material for: Association Mapping for Yield Attributing Traits and Yellow Mosaic Disease Resistance in Mung Bean [Vigna radiata (L.) Wilczek]
Source: Front Plant Sci. 2022 Jan 17;12:749439. doi: 10.3389/fpls.2021.749439 (PMC8801447; doi:10.3389/fpls.2021.749439)
Supplement: Supplementary file 3 [file Table_3.pdf]

Supplementary Table 3 Estimates of mean, range, PCV, GCV, heritability and genetic advance for studied traits in mungbean

**Traits**	**Mean**	**Range**	**PCV (%)**	**GCV (%)**	**Heritability (BS) (%)**	**Genetic advance**	**Gen. Adv per cent of Mean**
Days to 50% flowering	38	35-42	4.03	3.46	73.74	2.34	6.13
Days to maturity	64	56-68	3.95	3.81	92.98	4.92	7.56
Plant height (cm)	49	36-63	10.92	9.94	82.92	9.13	18.65
Pod length (cm)	7.42	6.3-9.5	8.13	7.69	89.58	1.11	15
Seed size (100-seeds weight) (g)	4.15	3.42-6.04	12.34	12.2	97.69	1.03	24.84
Yellow mosaic disease (1-9)	3.5	02-Jul	43.39	41.03	89.36	2.82	79.89
Number of branches per plant	2.95	2.2-3.4	22.05	21.68	96.66	1.67	43.91
Numer of pods per plant	23	18-27	9.91	8.77	78.38	3.74	16.01
Number of seeds per pod	9.8	9-11.2	4.37	3.55	65.95	0.58	5.94
Seed yield per plant (g)	9.83	7.03-12.06	9.9	9.19	86.3	1.73	17.6
