# Supplementary material for: Association Mapping for Yield Attributing Traits and Yellow Mosaic Disease Resistance in Mung Bean [Vigna radiata (L.) Wilczek]
Source: Front Plant Sci. 2022 Jan 17;12:749439. doi: 10.3389/fpls.2021.749439 (PMC8801447; doi:10.3389/fpls.2021.749439)
Supplement: Supplementary file 4 [file Table_4.pdf]

Supplementary Table 4a Analysis of variance for various quantitative characters of mungbean in *Kharif* (2018 & 2019 pooled)

**Source**	**df**	**Days to 50% flowering**	**Days to maturity**	**Plant height**	**Pod length**	**Seed size (100-seeds weight)**	**Yellow mosaic disease**	**Number of branches per plant**	**Number of pods per plant**	**Number of seeds per pod**	**Seed yield per plant**
Replication	1	1.406	0.225	19.6	0.306	0.346	0.225	0.249	1.806	0.09	0.288
Genotype	79	4.060**	16.672**	84.024**	0.956**	0.417**	5.765**	0.193**	13.930**	0.513**	4.000**
Error	79	0.659	0.833	10.689	0.079	0.014	0.314	0.087	2.021	0.137	0.323
C.D		1.619	1.82	6.52	0.56	0.237	1.117	0.587	2.835	0.738	1.134
C.V (%)		2.144	1.381	6.254	3.89	3.091	12.174	9.856	6.324	3.875	5.931

**Significant (p≤0.01); df: degrees of freedom; C.D: Critical difference; C.V: Coefficient of variation

Supplementary Table 4b Analysis of variance for various quantitative characters of mungbean in Summer (2019 & 2020 pooled)

**Source**	**df**	**Days to 50% flowering**	**Days to maturity**	**Plant height**	**Pod length**	**Seed size (100-seeds weight)**	**Yellow mosaic disease**	**Number of branches per plant**	**Numer of pods per plant**	**Number of seeds per pod**	**Seed yield per plant**
Replication	1	1.6	0.506	8.1	0.073	0.011	0.306	0.072	9.506	0.889	0.63
Genotype	79	6.352**	10.386**	44.337**	0.545**	0.727**	3.385**	0.183**	8.604**	0.276**	0.983**
Error	79	1.182	0.937	8.239	0.058	0.015	0.268	0.075	2.202	0.079	0.222
C.D		2.168	1.93	5.724	0.48	0.248	1.033	0.544	2.96	0.561	0.94
C.V (%)		2.803	1.51	6.281	3.161	2.79	17.066	9.334	6.074	2.781	4.678

**Significant (p≤0.01); df: degrees of freedom; C.D: Critical difference; C.V: Coefficient of variation

Supplementary Table 4c Analysis of variance for various quantitative characters of mungbean in *Kharif* and Summer (pooled over environment**s)**

**Source**	**df**	**Days to 50% flowering**	**Days to maturity**	**Plant height**	**Pod length**	**Seed size (100-seeds weight)**	**Yellow mosaic disease**	**Number of branches per plant**	**Number of pods per plant**	**Number of seeds per pod**	**Seed yield per plant**
Replication	1	0.3	0.006	0.62	0.014	0.12	0.75	0.18	0.15	0.29	0.41
Genotype	79	4.12**	12.71**	52.25**	0.69**	0.51**	4.45**	0.14**	9.54**	0.30**	1.76**
Error	79	0.62	0.46	4.87	0.038	0.006	0.25	0.042	1.16	0.06	0.13
C.D		1.54	1.35	4.4	0.38	0.15	0.99	0.45	2.14	0.5	0.71
C.V (%)		2.06	2.64	6.51	4.62	3.88	14.15	7.75	5.6	2.85	5.66

**Significant ($p \leq 0.01$); df: degrees of freedom; C.D: Critical difference; C.V: Coefficient of variation
