# Supplementary material for: Association Mapping for Yield Attributing Traits and Yellow Mosaic Disease Resistance in Mung Bean [Vigna radiata (L.) Wilczek]
Source: Front Plant Sci. 2022 Jan 17;12:749439. doi: 10.3389/fpls.2021.749439 (PMC8801447; doi:10.3389/fpls.2021.749439)
Supplement: Supplementary file 5 [file Table_5.pdf]

Supplementary Table 5 Significant marker-trait association identified from GLM approach in different environments

***Kharif*-2018**				***Kharif*-2019**				***Kharif*-Pooled**			
**Trait**	**Locus**	**p value**	**R^2**	**Trait**	**Locus**	**p value**	**R^2**	**Trait**	**Locus**	**p value**	**R^2**
DM	DMSSR001	0.00034	19.22	NP	BMd12	1.32E-05	32.42	NP	BMd12	3E-06	36.05
DM	BM212	0.000426	18.78	SW	VR021	3.2E-06	27.57	PH	CP1038	0.0001	23.34
NP	BMd12	1.58E-07	41.55	SW	BMd35	2.82E-05	27.80	PH	BMd23	0.0004	18.90
PH	CP1038	0.000156	19.46	SW	BMd12	6.14E-05	29.46	PL	CEDG97	0.0005	18.90
PH	DMSSR043	0.000284	14.50	DF	AF35050	0.0005	15.99	SW	VR021	4E-06	26.93
PH	BMd23	0.0005	15.66	DM	BM146	9.23E-05	20.97	SW	BMd35	3E-06	27.29
PL	CEDG97	0.00017	21.08	PH	CEDG97	3.18E-05	26.79	SW	BMd12	9E-05	28.50
PL	DMSSR001	0.000302	19.95	PH	CEDGAT009	0.000272	24.81	YMD	BMd12	0.0005	22.73
SW	VR021	9.31E-06	25.19	PH	CP10667	0.000356	21.72	PL	BMd35	0.0002	17.64
SW	BMd35	9.55E-06	25.14	PL	BMd35	4.24E-05	21.18	PL	CEDG24	0.0002	14.45
SW	BMd12	0.000245	26.17	PL	CEDG24	0.000142	15.69	YMD	CP1038	4E-05	25.52
YMD	BMd12	0.000174	24.66	PL	VR021	0.000305	17.47	NS	CP5096	0.0003	15.06
YMD	CP5096	0.000176	14.79	YMD	CP1038	4.71E-06	30.42	***Kharif* and Summer-Pooled**			
YMD	BM146	0.000441	24.73	**Summer-2020**				NP	BMd12	1E-05	33.34
**Summer-2019**				YMD	BMd12	0.00037	22.12	SW	VR021	0.0002	18.44
YMD	BMd12	5.45E-06	29.72	YMD	CP1038	8.78E-06	26.71	SW	BMd35	0.0002	18.76
YMD	CP5096	0.000173	14.27	**Summer-Pooled**				YMD	BMd12	5E-05	25.50
PL	BMd35	5.5E-09	36.86	YMD	BMd12	0.000101	24.81	YMD	CP5096	0.0003	12.81
PL	CEDG24	1.96E-08	34.94	YMD	CP5096	0.000314	13.27	PL	BMd35	5E-05	20.70
YMD	CP1038	8.92E-06	26.95	PL	BMd35	2.21E-05	22.9	PL	CEDG24	0.0005	13.16
NP	DQ9393	0.000108	17.19	PL	VR021	3.68E-05	21.95	PL	VR021	0.0002	17.87
PL	BMd12	7.04E-07	36.41	YMD	CP1038	1.04E-06	30.44	YMD	CP1038	2E-06	28.74
SY	CEDG220	0.000379	25.32	YMD	BMD26	0.000207	17	NP	DQ9393	0.0003	15.24
YMD	BMD26	0.000496	15.38	NS	CEDG97	0.000235	20.74	SY	CP1038	0.0005	20.97

DF: days to flowering, DM: days to maturity, PH: plant height (cm), PL: pod length, SW: 100-seed weight (g), YMD: Yellow mosaic disease, NB: number of branches per plant, NP: number of pods per plant, NS: number of seeds per pod, SY: seed yield per plant (g). p- value-corrected p-value based on Benjamini Hochberg 1995 where n is 89 and alpha is 0.05)
