# Supplementary material for: Association Mapping for Yield Attributing Traits and Yellow Mosaic Disease Resistance in Mung Bean [Vigna radiata (L.) Wilczek]
Source: Front Plant Sci. 2022 Jan 17;12:749439. doi: 10.3389/fpls.2021.749439 (PMC8801447; doi:10.3389/fpls.2021.749439)
Supplement: Supplementary file 6 [file Table_6.pdf]

Supplementary Table 6 List of significant marker-trait associations and QTLs related to yellow mosaic disease and yield attributing traits in mungbean reported in earlier studies

SSR and SNPs	Traits	R^2	References
VrD1	$MYMIV, SW, NP$	6.0, 8.12, 5.12	Singh et al. (2018)
CEDG228	$MYMIV$	8.0	
STSbr1	$MYMIV$	18.0	
CEDG044	$MYMIV, SW$	11.33, 8.11	
CEDG256	SW	8.0	
cp05325	SW	9.44	
GMES0214	SW, NS	12.55, 6.49	
CEDG166	NP	6.0	
MBSSR238	NP	11.80	
CEDG056	NS	7.12	
CEDG293	$MYMIV$	13.26	Singh et al. (2020)
CEDG225		2.43	
CEDG050		2.25	
CEDC139		2.3	
DMB SSR008		11.54	
CEDG121		4.76	
CEDG211		5.09	
CEDG191		1.3	
Cp01038		1.63	
DMB-SSR059		12.45	
CEDG166		2.83	
BM212		2.48	
6201291	SCC	8.34	Noble et al. (2018)
6182037		6.35	
6160964		6.26	
6160927		7.77	
15676224		6.66	
6200936		5.66	
6157161		6.39	
6175778		6.47	
6175622		6.22	
Vradi07g01630	salt stress tolerance		Breria et al.(2020)
Vradi09g09510			
Vradi09g09600			
V1G08340	phosphorus tolerance		Reddy et al.(2020)
VRADI01G05520			
VRADI04G10750			
Vradi01g00820	K and P concentrations		Wu et al. (2020)
Vradi01g00830			
Vradi01g00840			
Vradi05g16350			
Vradi07g26320	P concentration		
Vradi07g26340			
Vradi07g14180	K concentrations		
Vradi08g22740			
Vradi08g17100			
Vradi06g09900	Iron		

Vradi06g10020	metal translocation		
Vradi01g05570	Zn		
Vradi07g05950			
Vradi07g06200			

MYMIV- Mungbean Yellow Mosaic India Virus; SW- 100-seed weight; NP- number of pods per plant; NS- number of seeds per pod; SCC- seed coat color; K- potassium; P- phosphorous; Zn- zinc
